# Association between dietary animal-derived branched-chain amino acids and sarcopenia in older adults: a cross-sectional study based on Chinese community

**DOI:** 10.3389/fnut.2025.1657426

**Published:** 2025-10-01

**Authors:** Tianfeng Zhang, Caiyan Zhang, Hongzhen Du, Yaping Chang, Kaijia Zhao, Hongmei Xue, Mingyue Liang, Zengning Li

**Affiliations:** ^1^School of Public Health, Hebei Medical University, Shijiazhuang, China; ^2^Department of Clinical Nutrition, The First Hospital of Hebei Medical University, Shijiazhuang, China; ^3^Hebei Province Key Laboratory of Nutrition and Health, Shijiazhuang, China

**Keywords:** sarcopenia, branched-chain amino acid, animal-derived, aging, dietary

## Abstract

**Background:**

The free-living dietary intake of branched-chain amino acids (BCAAs, including leucine, isoleucine, and valine) may have an impact on sarcopenia. This study aimed to compare the dietary BCAAs of animal sources related to sarcopenia in older individuals aged ≥ 55 years living in Chinese communities.

**Methods:**

We enrolled 367 older individuals (112 males and 255 females) aged over 55 years in six communities. Sarcopenia was diagnosed based on Asian Working Group for Sarcopenia (AWGS2019). The free-living dietary intake of BCAAs was evaluated by using a 64-item food frequency questionnaire (FFQ). Multivariable logistic regression models were applied to examine the association between BCAAs and sarcopenia.

**Results:**

The overall prevalence of sarcopenia was 20.7% (76 in 367). The mean daily energy intake, protein, fat, and BCAAs were significantly lower in the sarcopenia older adults than in the non-sarcopenia group (*p* < 0.05). Logistic regression analysis revealed that increased intake of leucine (OR: 0.121, 95% CI: 0.045–0.327, *p* < 0.001), isoleucine (OR: 0.160; 95% CI: 0.061–0.421, *p* < 0.001), and valine (OR: 0.202; 95% CI: 0.076–0.534, *p* = 0.001) were associated with the decrease risk of sarcopenia. When stratified by food sources, animal-derived BCAAs intake was significantly associated with sarcopenia in older adults (OR: 0.819; 95% CI: 0.675–0.995, *p* = 0.044). However, no such association was found for plant-derived BCAAs (OR: 0.903; 95% CI: 0.742–1.098, *p* = 0.305).

**Conclusion:**

High intake of dietary BCAAs was strongly associated with lower risk of sarcopenia in older adults. Animal-derived BCAAs intake may decrease the risk of sarcopenia in older adults, whereas no such effect was observed for plant-derived BCAAs.

## 1 Introduction

The world population is aging, and the proportion of older adults is expected to double from 12% in 2015 to 22% in 2050 (1). One of the leading causes of reduced independent living in older adults, sarcopenia is a geriatric syndrome characterized by progressive and widespread loss of skeletal muscle mass and function. It is strongly associated with an increased risk of falls, disability and death in older adults. According to the 2019 diagnostic criteria updated by the Asian Working Group for Sarcopenia (AWGS2019), the condition requires a comprehensive assessment of muscle mass, muscle strength (handgrip strength, HGS), and physical performance (Five-Times Sit-to-Stand Test, FTSST; gait speed, GS) (2). Globally, the prevalence of sarcopenia ranges from 10% to 27% in adults aged 65 years or older, rising to 50% in long-term care facilities (3). The associated healthcare costs account for 1.5%–2.0% of GDP in many countries (4).

Metabolism of branched-chain amino acids (BCAAs, including leucine, isoleucine, and valine) plays a crucial role in muscle health (5–7). BCAAs are metabolized primarily in skeletal muscle, which is the main source of energy during exercise and contributes to protein synthesis (8, 9). Oral supplement is the most common way of BCAAs intake, which plays an important role in improving Serum nutritional markers (prealbumin and retinol-binding protein), muscle strength, and body composition (10). The per-meal anabolic threshold of dietary amino acid intake is higher in older individuals (i.e., about 2.5 to 2.8 g leucine) in comparison with young adults (11, 12). Recent studies highlight that dysregulated protein metabolism is a key mechanism underlying sarcopenia, with BCAAs gaining attention for their role in activating the mTOR pathway to regulate muscle protein synthesis. Leucine, the most metabolically active BCAA, not only directly stimulates muscle anabolism but also suppresses the expression of proteolytic enzymes (13). Summarized data in a systematic review and meta-analysis revealed that whey protein, leucine, and vitamin D supplementation can increase appendicular muscle mass in patients with sarcopenia (14, 15).

Various dietary factors have been confirmed to be associated with sarcopenia (16, 17). A study of community-dwelling adults aged over 60 compared the relationship between dietary intake, indicators of inflammation, and sarcopenia, which revealed that high levels of leucine, methionine, threonine, histidine, aspartic acid, calcium, zinc, and vitamin C were associated with a lower risk of sarcopenia (18, 19). However, the relationship of dietary BCAAs intake and its food sources to sarcopenia in older adults remains unclear. Liu et al. (20) reported positive associations between total BCAAs, isoleucine, leucine, valine, and muscle mass and muscle strength among 108,017 participants. Another clinical trial found that supplementation with BCAAs combined with resistance exercise significantly enhanced muscle mass and strength. BCAAs are more effective in combating sarcopenia by reducing cortisol levels, promoting muscle repair, and creating a synergistic effect with resistance training. Additionally, animal experiments further reveal that moderate restriction of BCAAs intake improves metabolic health and prolongs lifespan, but overdose may trigger negative effects such as insulin resistance, suggesting the importance of dosage control (21).

However, the meta-analysis came to the opposite conclusion, which indicated that BCAA alone or PUFA were not effective in improving muscle health (9). Other studies also show that BCAA supplements do not enhance protein synthesis or improve muscle strength, mass or physical performance (22, 23). Dietary BCAAs may have been linked to adverse effects on healthy aging (24). Therefore, the prevention of sarcopenia in the older population through dietary guidance or intervention still requires further research. Thus, this study aimed to investigate the association of free-living dietary BCAAs and its food sources with sarcopenia among older adults living in Chinese communities.

## 2 Materials and methods

### 2.1 Study participants

This study was conducted from June 2021 to July 2022. We used whole cluster random sampling to collect data on adults aged 55–90 years from multiple Chinese communities and nursing facilities across Hebei Province, China. The study protocol was approved by the Ethics Committee of the First Hospital of Hebei Medical University (Trial Registration: Chinese Clinical Trial Registry ChiCTR2200061824). All subjects provided written informed consent before data collection. To ensure the consistency of the questionnaires, all investigators received standardized training before the trial.

The inclusion criteria were as follows: (1) Aged 55–90 years. (2) Ability to communicate without barriers. (3) Capable of independently performing actions such as “standing up and sitting down” and “walking” without external assistance. The exclusion criteria were as follows: (1) Physical disabilities. (2) Presence of specific diseases (including Congestive Heart Failure, Chronic Obstructive Pulmonary Disorder, Chronic Renal Failure, cancer, etc.). (3) Installed with a cardiac pacemaker. (4) The presence of severe cognitive impairment or inability to communicate. (5) Activity limitations due to injury. (6) Refusal to sign or inability to understand the informed consent form.

### 2.2 Assessment of dietary intake

To investigate the relationship between daily BCAAs intake and sarcopenia, we used the Recommended Nutrient Intake (RNI) to determine whether BCAAs intake of each subject was inadequate. RNIs for leucine, isoleucine, and valine are 39, 20, and 26 mg/kg, respectively according to the standards issued by Joint WHO/FAO/UNU Expert Consultation (25). We applied a 64-item food frequency questionnaire (FFQ) to assess the usual free-living dietary intakes of subjects. We primarily investigated the dietary intake of the subjects over the past 12 months, including 15 major food categories (refined grains, whole grains, fried foods, vegetables, fungi and algae, fruits, nuts, legumes and soy products, pickled foods, meat and eggs, seafood, dairy products, sugary beverages, alcohol, and drinking). We also investigated the household consumption of cooking oils and condiments to better estimate daily dietary intake.

All subjects were asked to answer the following questions regarding food consumption quantity: (1) Record the frequency of consumption over the past year, with units of: times/year, times/month, times/week, times/day; (2) Record the average amount consumed per instance. Daily intake of each food category is calculated as:


Daliy⁢Intake=Consumption⁢Frequency×Average⁢Amount⁢per

Instance/Frequency⁢of⁢Consumption

### 2.3 Diagnosis of sarcopenia

Body composition was determined by bioelectrical impedance analysis (BIA) using an InBody S10 analyzer (BioSpace Co., Ltd., South Korea), including skeletal muscle mass (SMM), appendicular skeletal muscle mass (ASM), body fat mass (BF), and phase angle (PhA), muscle-to-fat ratio (MFR), etc. We assess subjects’ muscle mass using the appendicular skeletal muscle mass index (ASM/Height^2^, ASMI), where low muscle mass is defined as <7.0 kg/m^2^ for males and <5.7 kg/m^2^ for females. All measurements were performed in the morning for all participants, and they were asked not to change their usual dietary pattern and daily physical activity and to refrain from vigorous activities the day before the trial started.

The diagnosis of sarcopenia for the included participants is conducted according to the AWGS2019 guidelines (2). In that guideline, sarcopenia has been defined as the existence of both low muscle mass and low muscular strength or low physical performance.

### 2.4 Assessment of muscle strength

All subjects were asked to place their feet flat on the ground and muscle strength was assessed by testing HGS. The test arm should hang down, with the forearm positioned at an angle of no more than 15° from the body. The angle of wrist flexion is 0–30° and the upper arm is pressed against the chest. HGS was measured twice for each hand (once for each right and left hand), with a 20 s interval between measurements. We used the maximum value obtained in each measurement as the subject’s HGS value. AWGS2019 recommends the following diagnostic thresholds for sarcopenia based on HGS measurements: for males, < 28.0 kg; for females, < 18.0 kg.

### 2.5 Assessment of physical function

The Five-Times Sit-to-Stand Test (FTSST) was used to evaluate the physical function. Subjects were asked to sit on a chair with a height of 46 cm and no armrests, with both feet flat on the ground and hands crossed over the chest or placed on the shoulders. When the tester gives the “start” command, the subjects must stand up and sit down as quickly as possible without using their arms for support, completing five repetitions. The test ends after the fifth repetition when the participant’s body makes contact with the chair, and the overall time taken for the test is recorded. A total test time of >12 s is considered the cutoff value indicating a decline in physical function.

GS was measured over a 6 m straight walking course using a stopwatch. All subjects were asked to walk a distance of 6 m at a normal GS. The measurement was repeated twice, and the physical function was evaluated using the faster value as the evaluation index. A GS <1 m/s was determined to indicate low physical function.

### 2.6 Amino acids measurements

Blood samples were drawn after overnight fasting. Clinical and blood biochemical assessments was measured by Blood Cell Analyzer DxH800 Serie and Coulter Chemistry analyzer AU5800 Serie (UniCel, United States). Blood concentration for amino acid was measured using high-performance liquid chromatography and AccQ Tag Fluor Reagent Kit (Waters, United States).

### 2.7 Assessment of other variables

We also used the Mini Nutritional Assessment (MNA) scale to evaluate the nutritional status of the subjects. The Montreal Cognitive Assessment (MoCA) scale was employed to assess the cognitive function of the subjects, and the mobility was evaluated using the Activities of Daily Living Assessment Scale (ADL) as well. Required data on other variables such as demographic characteristics (including age, gender, education, and occupation), past medical history and medication use, alcohol intake, and smoking status were collected via pretested questionnaires.

### 2.8 Statistical analysis

Continuous variables are presented as means and standard deviations (SD) or median (inter-quartile range, IQR). The independent-sample *t*-test or Wilcoxon rank-sum test was used for comparisons. We used linear regression to analyze factors that may affect ASM in aged people.

To examine the association between BCAA intake and sarcopenia, we used binary logistic regression in three different models:

Model 1: adjusted for age, sex, daily energy intake.
Model 2: as Model 1 and adjusted for BMI.
Model 3: as Model 2 and adjusted for locations of communi-ties, average income, smoke and MOCA scores.

The first tertile (T1) of BCAA intake was defined as the reference category and odds ratios (OR) and 95% Confidence intervals (CIs) in the second/third tertiles (T2/T3) were computed. A two-tailed *p*-value of less than 0.05 was considered statistically significant between T1 and T3. Binary logistic regression models were used and adjusted for age, BMI, gender and food sources when used to explore the association between food-based BCAAs and sarcopenia in older adults.

## 3 Results

### 3.1 Characteristics of the participants

A total of 367 subjects participated in the study, including 112 males and 255 females aged over 55. The baseline characteristics of study subjects were provided in Table 1. The overall prevalence of sarcopenia was 20.7% (76 in 367), with no significant difference (*p* = 0.539) between males (18.8%, 21 in 112) and females (21.6%, 55 in 255). There were no significant differences in age, BMI or HC between males and females, but body fat mass was higher in females and muscle strength and quality indicators was higher in males (*p* < 0.001).

**TABLE 1:** Baseline characteristics of study participants.

Characteristics	Total (*n* = 367)	Male (*n* = 112)	Female(n = 255)	*P*
Sarcopenia	76	21	55	
Non-sarcopenia	291	91	200	0.539
Age (y)	65.53 ± 6.23	65.48 ± 5.60	65.56 ± 6.46	0.904
BMI (kg)	25.40 ± 3.61	25.23 ± 3.40	25.48 ± 3.7	0.511
ASMI (kg/m^2^)	7.14 ± 6.23	8.00 ± 0.92	6.78 ± 0.82	< 0.001
HGS (kg)	27.55 ± 8.61	35.59 ± 7.61	24.04 ± 6.39	< 0.001
SMM (kg)	24.78 ± 4.88	29.55 ± 4.54	22.71 ± 3.34	< 0.001
BF (kg)	21.28 ± 7.48	18.39 ± 6.82	22.53 ± 7.41	< 0.001
PhA (°)	5.16 ± 0.72	5.48 ± 0.70	5.03 ± 0.69	< 0.001
MFR	1.41 ± 1.24	2.01 ± 1.72	1.15 ± 0.84	< 0.001
CC (cm)	34.50 ± 3.46	35.11 ± 3.84	34.23 ± 3.25	0.014
WC (cm)	87.97 ± 13.67	92.25 ± 9.85	86.13 ± 14.65	< 0.001
HC (cm)	98.43 ± 9.36	98.10 ± 6.68	98.58 ± 6.68	0.621
Income	3074.77 ± 1659.84	3330.60 ± 1821.39	2963.47 ± 1574.62	0.032

BMI, body mass index; HGS, handgrip strength; SMM, skeletal muscle mass; BF, body fat mass; PhA, phase angle; CC: calf circumference; WC, waist circumference; HC, hip circumference; ASMI, appendicular skeletal muscle mass index; MFR, muscle-to-fat ratio

### 3.2 Characteristics between sarcopenia and non-sarcopenia groups

Table 2 showed that the sarcopenia group had higher averages in age, BMI, HGS, and SMM than the non-sarcopenia group. The mean daily energy intake, protein, fat, and BCAAs (including leucine, isoleucine, and valine) were significantly lower in the sarcopenia group than in the non-sarcopenia. The daily protein and fat intake in the sarcopenia group was higher than that in the non-sarcopenia group (*p* < 0.001), and there was no statistically significant difference in carbohydrate intake (*p* = 0.600). The non-sarcopenia group had higher per capita income (*p* < 0.001), MFR (*p* = 0.023) and better cognitive functioning (*p* < 0.001). However, we did not find significant differences in the serum indicators.

**TABLE 2:** Differences between sarcopenia and non-sarcopenia groups.

Variables	Non-sarcopenia (*n* = 291)	Sarcopenia (*n* = 76)	*P*
**General information**			
Age (y)	64.45 ± 6.08	67.63 ± 5.99	< 0.001
BMI (kg/m^2^)	25.94 ± 3.48	24.34 ± 3.63	< 0.001
PhA (°)	5.37 ± 0.70	4.76 ± 0.59	< 0.001
SMM (kg)	26.06 ± 4.96	22.25 ± 3.59	< 0.001
BF (kg)	21.48 ± 7.54	20.89 ± 7.36	0.455
MFR	1.51 ± 1.46	1.22 ± 0.56	0.023
HGS (kg)	29.93 ± 8.31	22.93 ± 7.21	< 0.001
MoCA	23.46 ± 4.00	20.64 ± 5.64	< 0.001
Income	3328 ± 1823	2592 ± 1125	< 0.001
**Dietary intake/d**			
Energy (kcal)	2070 ± 658	1875 ± 641	0.003
Carbohydrate (g)	305.81 ± 108.32	299.99 ± 115.24	0.600
Protein (g)	89.52 ± 29.23	76.38 ± 25.58	< 0.001
Fat (g)	57.02 ± 27.40	43.91 ± 20.72	< 0.001
Leucine (g)	1.93 ± 0.64	1.63 ± 0.53	< 0.001
Isoleucine (g)	3.56 ± 1.19	3.00 ± 0.98	< 0.001
Valine (g)	4.21 ± 1.48	3.72 ± 1.57	< 0.001
Refined grains (g)	2415.12 ± 1307.12	2513.68 ± 1349.04	0.457
Whole grains (g)	1202.08 ± 898.62	1039.79 ± 738.88	0.056
Vegetables (g)	2988.23 ± 2050.46	2667.09 ± 1949.95	0.112
Meats (g)	538.18 ± 668.45	297.82 ± 249.84	< 0.001
Serum*			
WBC (10^∧9^/L)	5.74 ± 1.49	5.82 ± 1.43	0.607
CRP (mg/L)	2.69 ± 4.03	2.57 ± 2.50	0.745
Insulin (μIU/mL)	7.93 ± 6.55	9.06 ± 11.06	0.710
Leu (mmol/L)	0.16 (0.06)	0.16 (0.06)	0.773
Ile (mmol/L)	0.08 (0.04)	0.08 (0.03)	0.952
Val (mmol/L)	0.24 (0.10)	0.26 (0.11)	0.075

*Sixty-four sarcopenia and 195 non-sarcopenia patients were included. BMI, body mass index; HGS, handgrip strength; PhA, phase angle; SMM, skeletal muscle mass; BF, body fat mass; MoCA, Montreal Cognitive Assessment; MFR, muscle-to-fat ratio; Leu, leucine; Ile, isoleucine; Val, valine.

### 3.3 Pearson correlation analysis between dietary intake, body composition, and serum BCAAs

**FIGURE 1:**
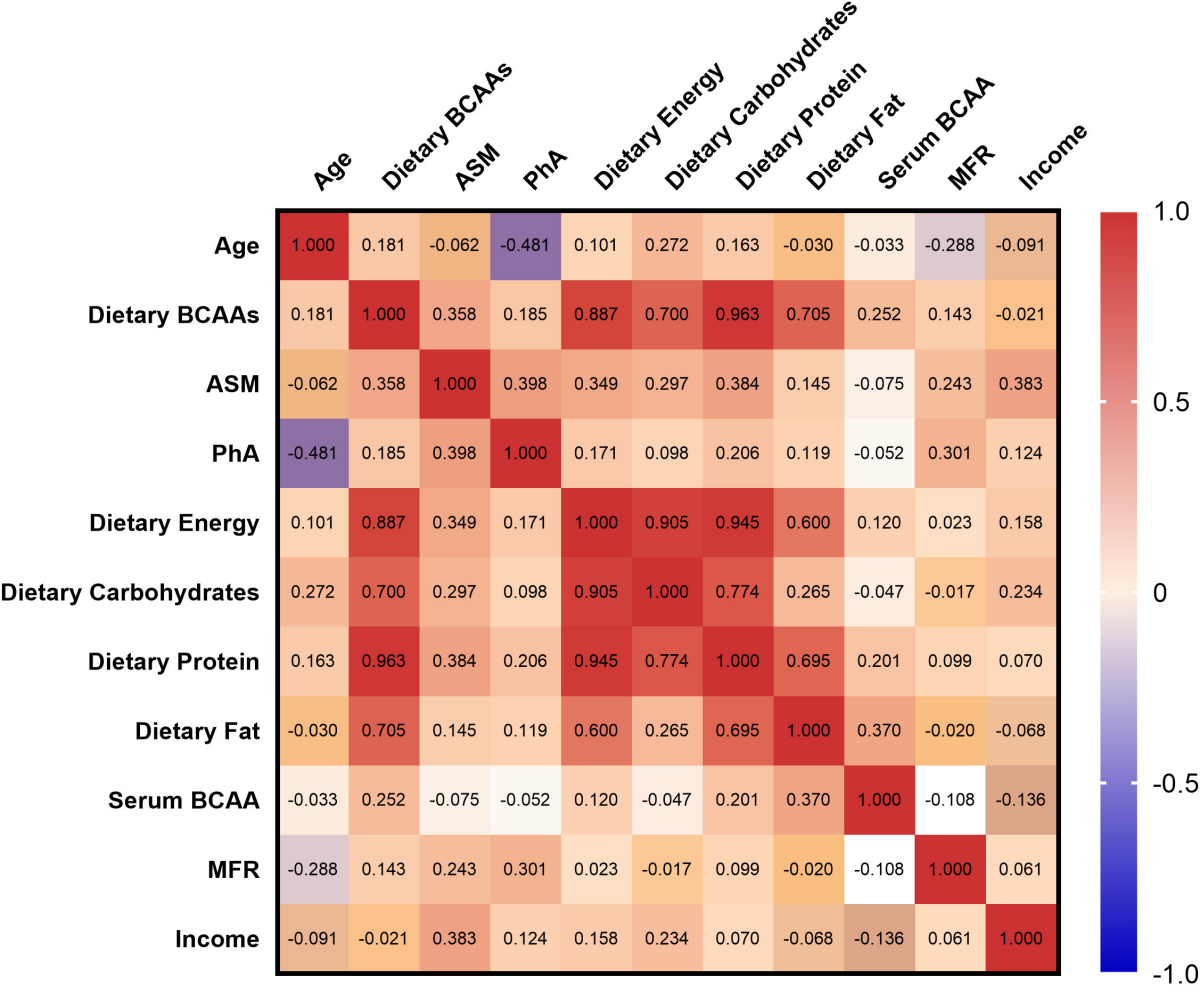
Spearman heatmap of anthropometric, nutritional, and metabolic parameters.

Pearson correlation analysis was performed to study the association between dietary intake, body composition, and serum BCAAs in sarcopenia adults in Figure 1. Dietary carbohydrate (r = 0.297, *p* = 0.011) and protein (r = 0.384, *p* < 0.001) intakes were related to ASM in the elderly, and the intake of dietary BCAAs showed a positive trend of ASM (r = 0.358, *p* = 0.002). However, serum BCAAs and dietary fat intake were more correlated (r = 0.370, *p* = 0.003), higher than dietary protein and carbohydrates intake (r = 0.201, *p* = 0.114; r = −0.047, *p* = 0.715).

### 3.4 The linear regression of ASM and other variables

The linear regression in Table 3 showed that, variables such as age, BMI, income per capita, and smoking may have influences on ASM in older adults, The prevalence of sarcopenia is associated with a lower intake of total energy, dietary fiber, protein, and BCAAs in the diet. We also observed an association between the prevalence of sarcopenia and low carbohydrate intake by adjusting for energy intake. Dietary factors were observed to have a large effect on muscle mass, strength, and mobility in older adults. After adjuested for age, gender, BMI and energy intake, dietary carbohydrate intake negatively correlated with muscle mass in older adults.

**TABLE 3:** Linear regression analysis affecting appendicular skeletal muscle mass (ASM).

Variables	β	*P*	95% CI
**Crued**			
Age (y)	−0.142	0.001	−0.133, −0.043
Phase angle (°)	0.503	< 0.001	2.366, 3.046
BMR (kcal)	0.951	< 0.001	0.020, 0.021
Income	0.116	< 0.001	0.170, 0.597
Smoke	0.472	< 0.001	3.980, 5.242
ADL	0.104	0.005	0.033, 0.188
**Model 1**			
BMI (kg/m^2^)	0.395	< 0.001	0.381, 0.475
BMR (kcal)	0.872	< 0.001	0.018, 0.020
**Model 2**			
Fiber (g)	0.108	0.001	0.023, 0.098
Energy (kcal)	0.250	< 0.001	0.001, 0.002
**Model 3**			
Carbohydrate (g)	−0.304	< 0.001	−0.015, −0.007
Protein (g)	0.200	0.014	0.006, 0.049
Fat (g)	0.022	0.615	−0.009, 0.015
Leucine (g)	0.090	0.005	0.289, 1.655
Isoleucine (g)	0.185	< 0.001	0.939, 3.162
Valine (g)	0.117	0.044	0.020, 1.529

Model 1: adjusted for age and gender. Model 2: as Model 1 and additionally adjusted for body mass index (BMI). Model 3: as Model 2 and additionally adjusted for energy intake.

### 3.5 Association of dietary BCAAs with sarcopenia and its components

The compliance rate for daily BCAAs intake (including leucine, isoleucine, and valine) among all subjects was 19.02%, 26.43%, and 17.44% (70 in 367; 97 in 367; 64 in 367), respectively. Binary logistic regression was used in three different models. The first tertile was defined as the reference category and ORs and 95% CIs in the second and third tertiles were computed. Based on the tertiles of the intake levels of the BCAAs, the subjects were divided into the lowest intake group (T1, *n* = 122), the moderate intake group (T2, *n* = 122), and the highest intake group (T3, *n* = 123).

Crude and multivariable-adjusted ORs and 95% CIs for sarcopenia and its components by tertiles of dietary leucine, isoleucine and valine are shown in Table 4. BCAAs intake in T3 were less likely to be sarcopenia than those in T1 in crude model (OR: 0.250, 95% CI: 0.148–0.425; OR: 0.271, 95% CI: 0.161–0.454; OR: 0.307, 95% CI: 0.184–0.511, *p* < 0.001). After adjustment for age, sex and daily energy intake, the association strengthened (OR: 0.115, 95% CI: 0.043–0.308, *p* < 0.001; OR: 0.153; 95% CI: 0.059–0.394, *p* < 0.001; OR: 0.190; 95% CI: 0.073–0.495, *p* = 0.001, respectively). Further adjustment for other potential confounders did not alter the association (Model 2, Model 3). With the increasing intake of BCAAs, the components of sarcopenia, including low HGS and low GS, also showed significant association.

**TABLE 4:** Multivariate adjusted odds ratio for sarcopenia and its components across tertiles of dietary branched-chain amino acids (BCAAs).

Models	Tertiles of leucine intake OR (95%CI)			*P* ^1^	Tertiles of isoleucine intake OR (95%CI)			*P* ^1^	Tertiles of valine intake OR (95%CI)			*P* ^1^
	T1 (*n* = 122)	T2 (*n* = 122)	T3 (*n* = 123)		T1 (*n* = 122)	T2 (*n* = 122)	T3 (*n* = 123)		T1 (*n* = 122)	T2 (*n* = 122)	T3 (*n* = 123)	
**Sarcopenia**												
Crude	1	0.556 (0.350–0.883)	0.250 (0.148–0.425)	< 0.001	1	0.499 (0.313–0.797)	0.271 (0.161–0.454)	< 0.001	1	0.514 (0.322–0.820)	0.307 (0.184–0.511)	< 0.001
Model 1	1	0.442 (0.254–0.766)	0.140 (0.059–0.334)	< 0.001	1	0.383 (0.220–0.668)	0.155 (0.066–0.363)	< 0.001	1	0.417 (0.239–0.727)	0.201 (0.086–0.470)	< 0.001
Model 2	1	0.405 (0.225–0.727)	0.116 (0.046–0.292)	< 0.001	1	0.355 (0.197–0.640)	0.122 (0.049–0.302)	< 0.001	1	0.381 (0.211–0.689)	0.161 (0.065–0.401)	< 0.001
Model 3	1	0.463 (0.247–0.865)	0.121 (0.045–0.327)	< 0.001	1	0.458 (0.242–0.866)	0.160 (0.061–0.421)	< 0.001	1	0.462 (0.245–0.875)	0.202 (0.076–0.534)	0.001
**Low HGS**												
Crude	1	0.490 (0.264–0.911)	0.304 (0.148–0.626)	0.001	1	0.514 (0.277–0.956)	0.312 (0.152–0.641)	0.002	1	0.443 (0.233–0.841)	0.371 (0.188–0.735)	0.004
Model 1	1	0.435 (0.206–0.917)	0.226 (0.071–0.722)	0.012	1	0.411 (0.194–0.869)	0.213 (0.066–0.689)	0.01	1	0.378 (0.176–0.811)	0.294 (0.093–0.930)	0.037
Model 2	1	0.403 (0.189–0.862)	0.182 (0.055–0.607)	0.006	1	0.381 (0.178–0.816)	0.168 (0.050–0.567)	0.004	1	0.350 (0.161–0.761)	0.232 (0.071–0.765)	0.016
Model 3	1	0.504 (0.204–1.242)	0.115 (0.022–0.614)	0.011	1	0.608 (0.241–1.534)	0.169 (0.033–0.874)	0.034	1	0.530 (0.210–1.337)	0.271 (0.057–1.281)	0.099
**Low GS**												
Crude	1	1.077 (0.684–1.694)	0.479 (0.293–0.786)	0.004	1	0.899 (0.570–1.419)	0.564 (0.349–0.911)	0.019	1	0.958 (0.608–1.509)	0.501 (0.308–0.816)	0.005
Model 1	1	0.823 (0.482–1.405)	0.224 (0.099–0.509)	< 0.001	1	0.733 (0.431–1.249)	0.340 (0.155–0.742)	0.007	1	0.690 (0.403–1.181)	0.228 (0.099–0.522)	< 0.001
Model 2	1	0.927 (0.536–1.605)	0.250 (0.108–0.580)	0.001	1	0.829 (0.481–1.431)	0.376 (0.168–0.839)	0.017	1	0.770 (0.443–1.337)	0.251 (0.107–0.588)	0.001
Model 3	1	0.947 (0.529–1.695)	0.222 (0.090–0.545)	0.001	1	0.836 (0.463–1.509)	0.325 (0.136–0.777)	0.011	1	0.733 (0.427–1.400)	0.200 (0.079–0.509)	0.001

^1^*P*-value between T3 and T1. Model 1: adjusted for age, sex, daily energy intake. Model 2: as Model 1 and adjusted for body mass index (BMI). Model 3: as Model 2 and adjusted for locations of communities, average income, smoke and Montreal Cognitive Assessment (MOCA) scores.

Table 5 shows the ORs for sarcopenia and dietary sources of BCAAs. In the crude model, dietary intake of animal-derived BCAAs was negatively associated with sarcopenia (OR: 0.766, 95% CI: 0.0.691–0.849, *p* < 0.001), whereas we did not observe an association between intake of plant-derived BCAAs and sarcopenia (OR: 0.952, 95% CI: 0.898–1.010, *p* = 0.101). This trend remained unchanged after further adjusted for age, gender, BMI and daily animal-based foods (OR: 0.819, 95% CI: 0.675–0.955, *p* = 0.044; OR: 0.903, 95% CI: 0.742–1.098, *p* = 0.305, respectively).

**TABLE 5:** Odds ratios for sarcopenia and dietary sources of branched-chain amino acids (BCAAs).

Variables	Crude		Adjusted model	
	OR (95%CI)	*P*	OR (95%CI)	*P*
**Animal-derived^1^**				
BCAAs	0.766 (0.691–0.849)	< 0.001	0.819 (0.675–0.995)	0.044
Leu	0.564 (0.452–0.704)	< 0.001	0.651 (0.429–0.990)	0.045
Lle	0.347 (0.231–0.522)	< 0.001	0.456 (0.212–0.983)	0.045
Val	0.390 (0.271–0.561)	< 0.001	0.493 (0.248–0.978)	0.043
**Plant-derived^2^**				
BCAAs	0.952 (0.898–1.010)	0.101	0.903 (0.742–1.098)	0.305
Leu	0.902 (0.797–1.020)	0.100	0.832 (0.572–1.209)	0.335
Lle	0.803 (0.629–1.026)	0.080	0.578 (0.256–1.303)	0.186
Val	0.857 (0.702–1.046)	0.129	0.732 (0.337–1.587)	0.429

^1^Model adjusted for age, gender, body mass index (BMI) and animal-based foods.

^2^Model adjusted for age, gender, BMI and plant-based foods.

## 4 Discussion

This study retrospectively investigated the free-living dietary intake of aged adults, and explored whether dietary BCAAs intake can influence the risk of sarcopenia. Insufficient BCAAs intake in aged people in Chinese communities was quite common. The mean daily energy intake, protein, fat, and BCAAs (including leucine, isoleucine, and valine) were significantly lower in the sarcopenia group than in the non-sarcopenia. When daily energy intake was maintained at a constant level, decreased carbohydrate intake and increased protein intake contributed to decreased muscle loss, and increased intake of all three BCAAs contributed to increased muscle strength and mobility in older adults. The findings also indicate that, higher animal-derived dietary BCAAs intake is correlated with a lower risk of sarcopenia in aged adults, whereas no such effect was observed for plant-derived BCAAs.

Branched-chain amino acids plays a significant role in the treatment and prevention of sarcopenia (26), and the insufficient BCAA intake among older adults have been reported (27). Bustos-Arriagada et al. (28) found that nearly 80% of Chilean elderly aged 60–80 had insufficient leucine intake, but the differences in leucine intake among the elderly were not significantly associated with BMI, grip strength, and muscle mass (28). Another study also suggests that while BCAAs supplementation can improve grip strength and physical activity levels, it cannot reverse sarcopenia caused by aging. Research in recent years has indicated that metabolic disorders of BCAAs are closely related to the development and progression of sarcopenia. Zuo et al. (29) revealed the molecular mechanisms by which BCAA metabolic disorders lead to skeletal muscle atrophy through RNA-seq and untargeted metabolomics analysis. Multi-omics analysis found that the disruption of BCAA catabolism is a major pathogenic mechanism of sarcopenia. In mouse models, the accumulation of BCAAs led to impaired skeletal muscle function, while enhancing BCAA catabolism through the drug BT2 significantly improved skeletal muscle function. This suggests that the reasonable intake of BCAAs and the promotion of their catabolism may be an effective strategy for the prevention and treatment of sarcopenia.

Based on the current food repertoire, the diet must always include sufficient amounts of some animal foods (namely seafood and dairy products) to meet the requirements for nutrients that become limiting as the percentage of plant protein increases: iodine, calcium, EPA + DHA, bioavailable iron, vitamin A, vitamin B12 and riboflavin (30). Besides, animal-drived proteins exhibit higher digestibility. Animal-drived proteins had higher amino acid bioaccessibility, while the compact structure of soybean protein led to decreased digestibility. These differences could be attributed to variations in the stability of the digestive system, primarily due to differences in protein secondary structure and amino acid charge (31). However, Ebrahimi-Mousavi et al. (22) have reported that there is no significant association between dietary intake of BCAAs and sarcopenia. This aforementioned conclusion is likely related to the age inclusion criteria of the study, which included younger subjects resulted in no statistically significant. Therefore, we limited the age of the population to 55–90 years, in order to explore dietary nutrient intake in the older adults. Our study separately investigated the roles of leucine, isoleucine, and valine intake across multiple models. Results showed that the increased BCAAs could effectively reduce the incidence of sarcopenia in older adults, with notable improvements in muscle strength and walking speed.

Branched-chain amino acids supplementation alone has shown variable effects, especially in sedentary individuals or when total protein intake is already sufficient (32). Resent study validation in both human cohorts and mouse models confirmed the critical role of BCAA catabolism in regulating sarcopenia (33, 34). Muscle-specific knockout of the PPM1K phosphatase reduces BCAA catabolism and was sufficient to drive accelerated muscle-aging phenotypes with reduced muscle mass and strength. It demonstrates that impaired BCAA catabolism in skeletal muscle contributes to the progression of sarcopenia (29). Recent studies have indicated a correlation between certain Amino acids and sarcopenia (6, 35). Zhan et al. (36) discovered potential causal relationships between amino acids and traits associated with sarcopenia, including Glutamine, Tyrosine, Glycine, and BCAAs, play positive roles in muscle metabolism (36). Another mediation analysis indicating that muscle mass potentially serves as a complete mediator in the pathway connecting total BCAA and valine to muscle strength. Additionally, it acts as a partial mediator in the pathway linking leucine to muscle strength and obscures the true effect of isoleucine on muscle strength (20). Besides, BCAAs not only affect the metabolism of skeletal muscle, but also have important impacts on the overall metabolism of the body and the gut microbiota (37). Future research should further explore the metabolic regulatory mechanisms of BCAAs, develop therapeutic strategies based on BCAAs, and assess their potential applications in clinical settings.

Nevertheless, this study has several limitations that must be acknowledged. First, the cross-sectional design of the study limits the ability to infer causal relationships between dietary intake, amino acid profiles, and the development of metabolic syndrome. Longitudinal studies would be needed to establish causal links. Second, the sample size, although adequate for the statistical analyses, may not be large enough to fully capture the diversity of dietary and outcomes.

## 5 Conclusion

Based on our results, high intake of dietary BCAAs could be strongly associated with the lower risk of sarcopenia in older adults. Higher animal-derived BCAAs may help prevent sarcopenia in older adults, whereas no such effect was observed for plant-derived BCAAs. These findings highlight that nutrients from different food sources may also be key factors in preventing sarcopenia. Nevertheless, our findings did not demonstrate a direct correlation between serum BCAA levels and sarcopenia. Further cohort or interventional studies are necessary to validate our findings.

## Data Availability

The data supporting the findings of this study can be obtained from the corresponding author upon a reasonable request. Requests for access to the dataset should be directed to zengningli@hebmu.edu.cn.
